# Retraction Notice: Onabotulinum toxin a treatment for posttraumatic trigeminal neuropathic pain: case series and literature review

**DOI:** 10.22514/jofph.2025.040

**Published:** 2025-06-12

**Authors:** Journal of Oral & Facial Pain and Headache Editorial Office

**Affiliations:** ^1^MRE Press, Far East Finance, 048545 Singapore, Singapore

**Keywords:** None.

## Abstract

No abstract available.

This article [1] has been retracted by the Editorial Office and the publisher, MRE Press. Following its publication, scholars raised concerns about the inclusion of identifiable patient images without an explicit statement confirming informed consent for their use in the article. The Editorial Office promptly contacted the authors for clarification and documentation.

The authors confirmed that informed consent had been obtained from the patients for the use of their images at the time of the study. However, they reported that retrieving the original consent forms was not possible due to significant logistical difficulties: the corresponding author had retired from the affiliated National Health Service (NHS) Trust, the co-authors had ended their affiliations with the involved institutions, and the relevant patient records had been partially digitized, restricting access.

The Editorial Office conducted a thorough review and considered alternatives, such as removing or further anonymizing the images. However, these options could not resolve the ethical issue, as the original publication did not include documented consent consistent with current standards. The absence of such documentation was found to contravene the journal’s ethical policies and international guidelines, including those of the International Committee of Medical Journal Editors (ICMJE) and the Committee on Publication Ethics (COPE). After careful deliberation, the decision was made to retract the article to uphold the principles of ethical publishing and patient privacy.

The full text and PDF of the article have been removed from this page, and this notice serves as the official record of its status.
